# In-Silico Multi-Omics Analysis of the Functional Significance of Calmodulin 1 in Multiple Cancers

**DOI:** 10.3389/fgene.2021.793508

**Published:** 2022-01-12

**Authors:** Maolin Yao, Lanyi Fu, Xuedong Liu, Dong Zheng

**Affiliations:** Laboratory of Genetics and Molecular Biology, College of Wildlife and Protected Area, Northeast Forestry University, Harbin, China

**Keywords:** multi-omics, calmodulin, prognosis analysis, immune infiltration, cancer biomarker

## Abstract

Aberrant activation of calmodulin 1 (*CALM1*) has been reported in human cancers. However, comprehensive understanding of the role of *CALM1* in most cancer types has remained unclear. We systematically analyzed the expression landscape, DNA methylation, gene alteration, immune infiltration, clinical relevance, and molecular pathway of *CALM1* in multiple cancers using various online tools, including The Cancer Genome Atlas, cBioPortal and the Human Protein Atlas databases. Kaplan–Meier and receiver operating characteristic (ROC) curves were plotted to explore the prognostic and diagnostic potential of *CALM1* expression. Multivariate analyses were used to evaluate whether the *CALM1* expression could be an independent risk factor. A nomogram predicting the overall survival (OS) of patients was developed, evaluated, and compared with the traditional Tumor-Node-Metastasis (TNM) model using decision curve analysis. R language was employed as the main tool for analysis and visualization. Results revealed *CALM1* to be highly expressed in most cancers, its expression being regulated by DNA methylation in multiple cancers. *CALM1* had a low mutation frequency (within 3%) and was associated with immune infiltration. We observed a substantial positive correlation between *CALM1* expression and macrophage and neutrophil infiltration levels in multiple cancers. Different mutational forms of *CALM1* hampered immune cell infiltration. Additionally, *CALM1* expression had high diagnostic and prognostic potential. Multivariate analyses revealed *CALM1* expression to be an independent risk factor for OS. Therefore, our newly developed nomogram had a higher clinical value than the TNM model. The concordance index, calibration curve, and time-dependent ROC curves of the nomogram exhibited excellent performance in terms of predicting the survival rate of patients. Moreover, elevated *CALM1* expression contributes to the activation of cancer-related pathways, such as the *WNT* and *MAPK* pathways. Overall, our findings improved our understanding of the function of *CALM1* in human cancers.

## Introduction

As one of the most common diseases worldwide, cancer threatens human life and public health. The pathogenesis of cancer is very complex and involves several cancer-critical genes, which control fundamental cell division and growth processes (Rezatabar et al., 2019; Lacroix et al., 2020). Therefore, it is important to perform multi-omics analysis for any cancer-critical gene, followed by further assessment of their molecular function in tumorigenesis and correlation of the same with clinical prognosis.

Calmodulin (CALM), the best-studied Ca^2+^-binding protein, is composed of 148 amino acids, and contains two globular domains linked by a highly flexible central linker domain (Ikura and Ames, 2006). A previous study had shown CALM to be a valuable peripheral biomarker for Alzheimer’s disease, discriminating the latter from other disorders related to dementia (Esteras et al., 2013). Another study showed that CALM expression significantly increases in cerebrospinal fluid of patients with Creutzfeldt Jakob disease (CJD) and might be used as a diagnostic biomarker for CJD (Chen et al., 2021). Moreover, the abnormally high expression of CALM can indicate liver fibrosis (Ji et al., 2019) and recurrence of nasopharyngeal carcinoma (Meng et al., 2017). In mammals, three genes encode CALM, namely *CALM1, CALM2,* and *CALM3* (Zhang et al., 2012). Although these genes only differ in their non-coding regions, they have distinct cellular functions, based on subcellular distribution and epigenetics (Toutenhoofd and Strehler, 2000). In this study, we specifically focused on *CALM1*. *CALM1* can regulate cell motility, differentiation, and proliferation (Chin and Means, 2000); increased *CALM1* expression had previously been detected in nasopharyngeal carcinoma (Zamanian Azodi et al., 2018), prostate cancer (Adeola et al., 2016), and bladder cancer (Zhang et al., 2018) and has been reported to play an oncogenic role in esophageal squamous cell carcinoma (Liu et al., 2021). However, our knowledge regarding the expression pattern, gene mutation, molecular function, and clinical value of *CALM1* and relationship of *CALM1* expression with DNA methylation and immune infiltration in most cancers is still lacking.

Here, we aimed to conduct comprehensive and systematic analyses of *CALM1* in human cancers. This study had five analysis modules, namely gene expression, DNA methylation and gene alterations, immune infiltration, prognostic and diagnostic potential, and relevant cellular pathways. We found that *CALM1* expression was upregulated in most cancer types and was closely related to immune infiltration and cancer-related cellular pathways. DNA methylation affected *CALM1* expression in multiple cancers. Additionally, *CALM1* expression had high diagnostic and prognostic potential. Taken together, our findings revealed that *CALM1* could be a promising prognostic and diagnostic biomarker for determining patient survival in human cancers.

## Materials and Methods

### Data Acquisition and Processing

We downloaded gene expression RNA-seq data of 33 The Cancer Genome Atlas (TCGA) cancer types and corresponding normal tissues from the Genotype-Tissue Expression database (GTEx; http://www.gtexportal.org/) and TCGA database (https://cancergenome.nih.gov/). These data were detected from 798 TCGA normal tissues, 9,807 TCGA cancer tissues, and 7,498 GTEx normal tissues, and were further processed using the Toil method (Vivian et al., 2017) and log2 transformed. Relevant clinical information (survival time, TNM stage, pathologic stage, etc.) of each patient and DNA methylation data (HM450) were also extracted from TCGA. Alteration frequency, mutation type and mutated site of *CALM1* in 33 TCGA cancer types were obtained from the cBioPortal database (https://www.cbioportal.org/). GSE41613 was downloaded from the GEO database (https://www.ncbi.nlm.nih.gov/geo/), including 97 HNSC samples with complete follow-up information (survival time and status). Additionally, we calculated the enrichment rate of immune cells in each cancer sample by the ssGSEA algorithm that used the specific markers (Bindea et al., 2013) of immune cell as a gene set to calculate the enrichment score of immune cells in each sample.

### Gene Expression Analysis

We compared the *CALM1* mRNA distribution between cancer tissues and adjacent non-cancer tissues in 33 TCGA cancer types. Owing to the abnormal distribution of *CALM1* expression values, we used the Mann–Whitney *U* test to compare *CALM1* expression between normal and cancer tissues. Subsequently, we investigated the correlation between *CALM1* expression and clinical features in different cancers using the Kruskal–Wallis test. Protein-wide omics data from the Human Protein Atlas (HPA; https://www.proteinatlas.org/) was used to verify the expression of *CALM1* between normal and cancer tissues. Analysis and visualization of data were based on the R language and a *p*-value of <0.05 indicated significance.

### DNA Methylation and Gene Alteration Analysis

We performed DNA methylation analysis using the transcriptome data of *CALM1* and DNA methylation data (HM450) from TCGA. Beta values were used to estimate the methylation levels of *CALM1* DNA. Differences in *CALM1* methylation between cancer tissues and adjacent non-cancer tissues were compared using the Mann–Whitney *U* test. The Pearson correlation method was used to measure the relationship between *CALM1* expression and methylation. A *p-*value of <0.05 and correlation coefficient of < −0.1 were regarded as the cut-off points. Subsequently, we conducted gene alteration analysis based on the cBioPortal database (Cerami et al., 2012). Genetic alterations of *CALM1* were obtained from the “Quick select” module. Results of alteration frequency and the mutation type of *CALM1* in human cancers were obtained using the “Cancer Type Summary” module. The 3D structure of the mutated site of *CALM1* was further observed with the “Mutations” module.

### Immune Infiltration Analysis

Using the ssGSEA algorithm in the R package GSVA (Sonja et al., 2013) and the transcriptome data of *CALM1* from TCGA, we investigated the correlation between *CALM1* expression and immune infiltration in human cancers. The Spearman correlation method was used to calculate the correlation coefficient. A total of four immune cells were included in this analysis, namely macrophages, B-cells, CD8+ T-cells, and neutrophils. Subsequently, we used the Tumor IMmune Estimation Resource 2 (TIMER2; http://timer.cistrome.org/) database (Li et al., 2020) to verify the correlation via the “Gene” module. Additionally, we investigated the correlation between immune infiltration abundance and Somatic Copy Number Alteration (SCNA) of *CALM1* using the “SCNA” module of the TIMER database (https://cistrome.shinyapps.io/timer/; (Li et al., 2017).

### Prognostic and Diagnostic Potential Analysis

We performed Kaplan–Meier (KM) analysis to assess the effect of CALM family genes expression on patient survival. Patients were divided into low- and high-expression groups according to the median expression of the CALM family genes. Differences in survival rates between the two groups were compared by Cox regression. Moreover, we used the GSE41613 dataset to verify the prognostic potential of *CALM1* expression in head and neck squamous cell carcinoma (HNSC). The University of Alabama Cancer database (UALCAN; http://ualcan.path.uab.edu/; Chandrashekar et al., 2017) was used to verify the prognostic potential of *CALM1* expression in liver hepatocellular carcinoma (LIHC). Subsequently, the diagnostic ROC curve (Do and Le, 2020; Do et al., 2021) was plotted, and the area under the curve (AUC) was calculated to define the diagnostic value of CALM family genes mRNA levels in cancers. The Cox proportional hazards regression model was used to evaluate whether *CALM1* expression could be a risk factor for overall survival (OS) in TCGA cancers. Using TCGA-HNSC data, we established a nomogram integrating *CALM1* expression, N stage, smoker, and radiation therapy for predicting patient survival rate at 1, 3, and 5 years. Performance of this nomogram was evaluated via concordance index (C-index), calibration curve, and time-dependent receiver operating characteristic (ROC) curves and compared with that of the traditional TNM nomogram via decision curve analysis (DCA).

To further assess the general applicability of the established model, we selected the TCGA-LIHC cohort as the validation set. Similarly, a nomogram integrating *CALM1* expression, TNM stage, pathologic stage and tumor status was developed, and its efficacy in predicting patient survival rate was evaluated using the C-index, calibration curve, ROC curves and DCA. Gene expression and clinicopathological information were extracted from TCGA. Analysis and visualization of data were accomplished using R language, and a *p*-value of <0.05 indicated significance.

### Insights Into *CALM1* Function

The Search Tool for the Retrieval of Interacting Genes/Proteins (STRING; https://string-db.org/) is commonly used to predict protein-protein interactions (Szklarczyk et al., 2019). According to this database, we screened 50 experimentally determined *CALM1*-binding proteins. Subsequently, we obtained 100 *CALM1*-correlated genes using the “Similar Gene Detection” module of the Gene Expression Profiling Interactive Analysis 2 (GEPIA2; http://gepia2.cancer-pku.cn/) database (Tang et al., 2019). Four genes with the strongest correlation with *CALM1* were screened for further expression analysis by the TIMER2 database. Finally, to elucidate functional differences for *CALM1*-high expression group versus *CALM1*-low expression group in HNSC and LIHC, we performed Gene Set Enrichment Analysis (GSEA) using the R package “clusterProfiler” (Yu et al., 2012). An adjusted *p-*value of <0.05 and FDR of <0.25 indicated significance.

## Results

### Gene Expression Analysis Data

**FIGURE 1:**
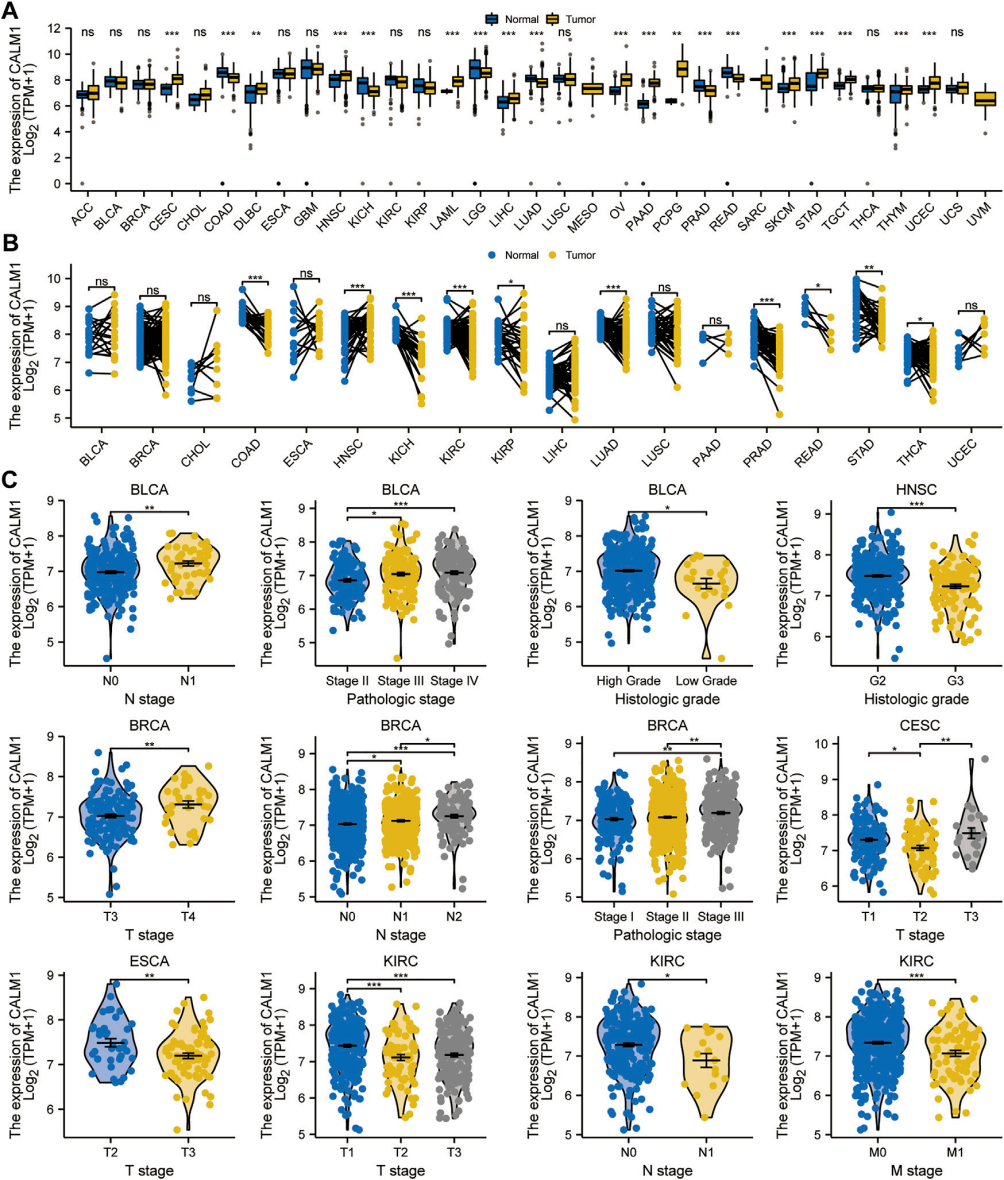
Expression levels of *CALM1* gene in human cancers and relevant clinical features. **(A, B)** Expression levels of *CALM1* mRNA in cancer and adjacent/paired normal tissues. *P < 0.05; **P < 0.01; ***P < 0.001; ns, no significance. **(C)** Relationship between *CALM1* mRNA expression and clinical features in human cancers. *P < 0.05; **P < 0.01; ***P < 0.001.

We assessed *CALM1* mRNA expression profiles in human cancers using data from the TCGA and GTEx databases. The results showed that *CALM1* mRNA expression is increased in 13 cancers, including the lymphoid neoplasm diffuse large B-cell lymphoma (DLBC; *P* = 0.002), LIHC (*P* < 0.001), cervical squamous cell carcinoma and endocervical adenocarcinoma (CESC; *P* < 0.001), HNSC (*P* < 0.001), acute myeloid leukemia (LAML; *P* < 0.001), ovarian serous cystadenocarcinoma (OV; *P* < 0.001), pancreatic adenocarcinoma (PAAD; *P* < 0.001), pheochromocytoma and paraganglioma (PCPG; *P* = 0.003), testicular germ cell tumor (TGCT; *P* < 0.001), stomach adenocarcinoma (STAD; *P* < 0.001), skin cutaneous melanoma (SKCM; *P* < 0.001), thymoma (THYM; *P* = 0.001), and uterine corpus endometrioid carcinoma (UCEC; *P* < 0.001), compared with that in normal tissues. However, *CALM1* mRNA expression is decreased in colon adenocarcinoma (COAD; *P* < 0.001), kidney chromophobe (KICH; *P* = 0.001), lower grade glioma (LGG; *P* < 0.001), lung adenocarcinoma (LUAD; *P* < 0.001), prostate adenocarcinoma (PRAD; *P* < 0.001), and rectum adenocarcinoma (READ; *P* < 0.001) (Figure 1A). The abbreviations and full names of TCGA cancers are listed in Supplementary Table S1. For paired normal and cancer tissues, the same difference in *CALM1* mRNA expression was verified in kidney renal papillary cell carcinoma (KIRC; *P* < 0.001), HNSC (*P* < 0.001), COAD (P < 0.001), KICH (*P* < 0.001), kidney clear cell carcinoma (KIRC; *P* < 0.001), LUAD (*P* < 0.001), PRAD (*P* < 0.001), READ (*P* = 0.031), STAD (*P* = 0.004), and thyroid carcinoma (THCA; P < 0.05) (Figure 1B). Overall, compared with non-cancer tissues, most cancer types had higher *CALM1* expression. We also observed a significant association between *CALM1* expression and clinical features (e.g., TNM stage, pathologic stage, and histologic grade) in human cancers (Figure 1C).

**FIGURE 2:**
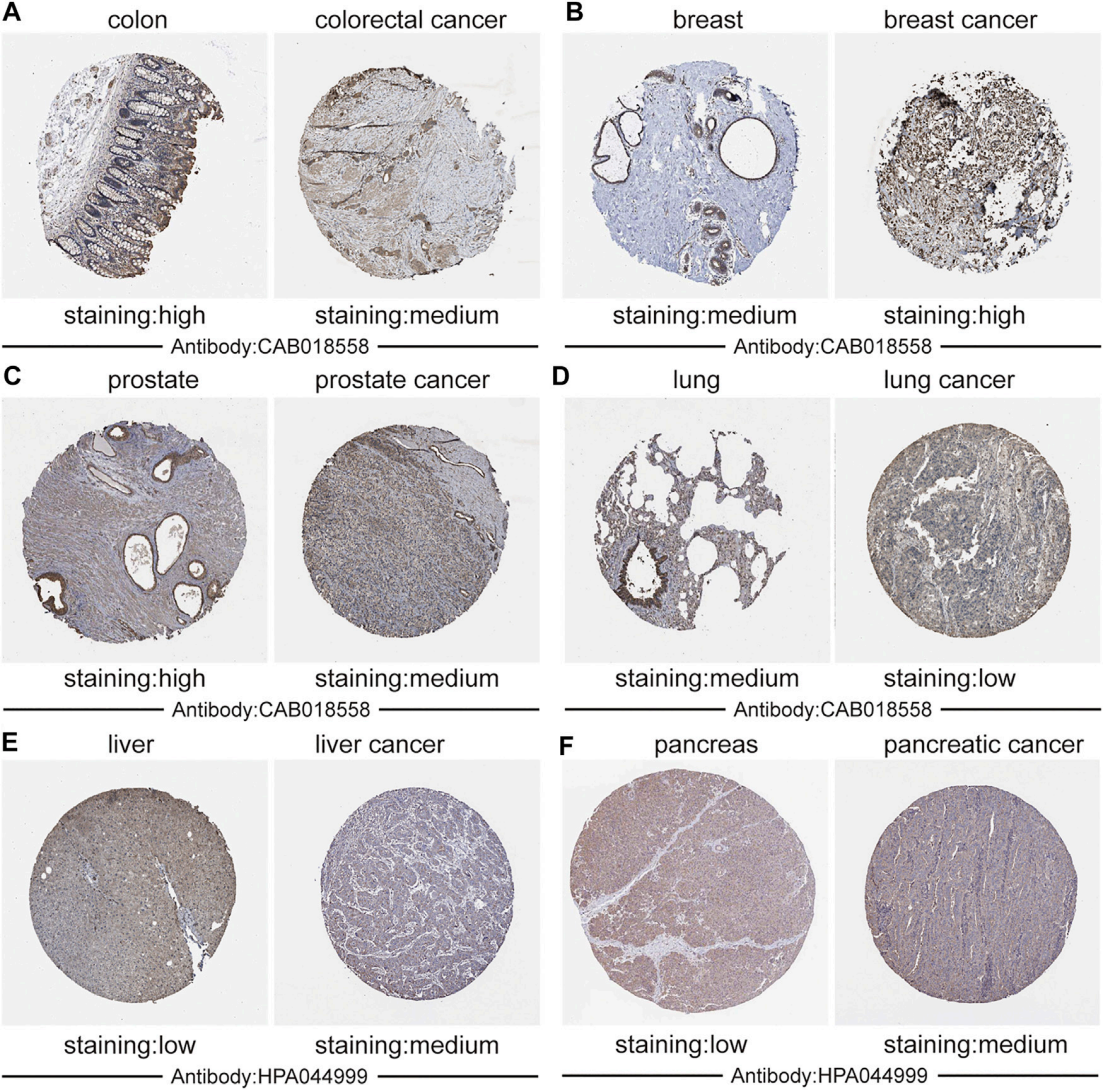
Validation the expression of *CALM1* on translational level using immunohistochemistry from the Human Protein Atlas database. **(A)** Colon and colorectal cancer. **(B)** Breast and breast cancer. **(C)** Prostate and Prostate cancer. **(D)** Lung and lung cancer. **(E)** Liver and liver cancer. **(F)** Pancreatic and pancreatic cancer. Staining: low, medium, and high. Antibody: CAB018558 and HPA044999.

Additionally, we used immunohistochemistry staining to verify *CALM1* expression in human cancers (Figure 2). The results showed that *CALM1* staining was increased in breast cancer, liver cancer, and pancreatic cancer but decreased in colorectal cancer, prostate cancer, and lung cancer, compared with that in normal tissues. These results demonstrated that the protein expression of *CALM1* increases in breast cancer, liver cancer, and pancreatic cancer, but decreases in colorectal cancer, prostate cancer, and lung cancer, compared with that in normal tissues, which is consistent with the mRNA levels of *CALM1*.

### DNA Methylation and Alteration Analysis Data

**FIGURE 3:**
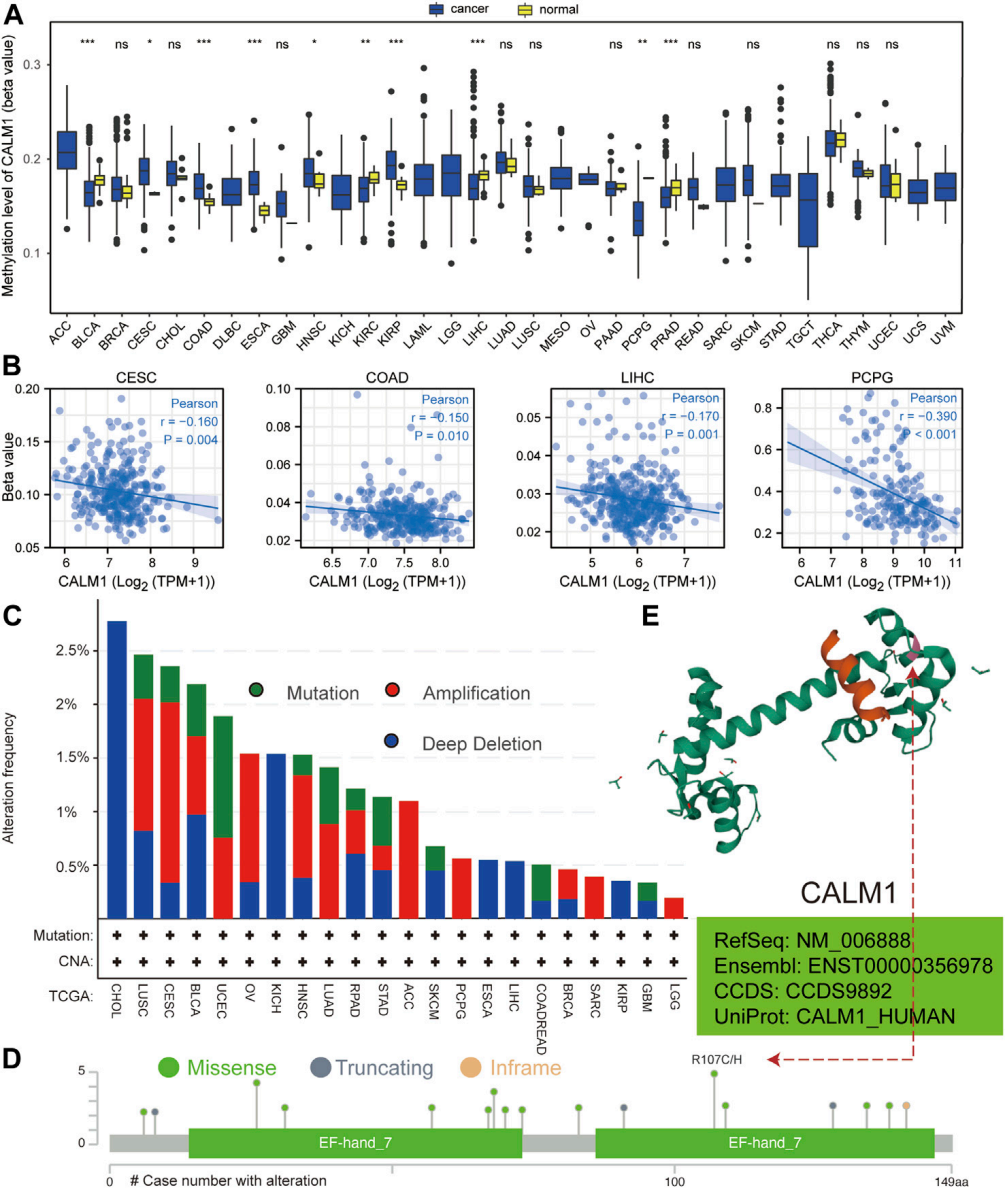
Analysis of DNA methylation and alteration of *CALM1* in human cancers. **(A)** Methylation level of *CALM1* in normal and cancer specimens. *P < 0.05; **P < 0.01; ***P < 0.001; ns, no significance. **(B)** Relationship between *CALM1* mRNA levels and DNA methylation in cervical and endocervical cancer (CESE), colon adenocarcinoma (COAD), liver hepatocellular carcinoma (LIHC), and pheochromocytoma and paraganglioma (PCPG). **(C)** Alteration frequency and mutation type of *CALM1* is displayed. **(D)** The types, sites, and case numbers of *CALM1* genetic alteration are presented. **(E)** The mutation site numbered 107 is displayed in the 3D structure of *CALM1* protein.

Based on the data from TCGA, we analyzed *CALM1* methylation levels between normal and cancer specimens. The results showed *CALM1* methylation to be significantly increased in cervical and endocervical cancer (CESC) and COAD compared with that in normal tissues, but significantly decreased in LIHC and PCPG (Figure 3A), showing an inverse trend compared with that of the expression pattern. Subsequently, we explored the relationship between *CALM1* mRNA expression and DNA methylation. Our results showed a negative association between DNA methylation and *CALM1* expression in four selected cancers (Figure 3B), which further demonstrates the regulatory ability of DNA methylation on *CALM1* expression. Additionally, DNA alteration analysis indicated that *CALM1* has a relatively low alteration rate, with the maximum not exceeding 3%. These alterations included missense mutations, amplification, and deep deletion; the adrenocortical carcinoma (ACC), PCPG, sarcoma (SARC), and LGG cases only carried amplification alteration of *CALM1* (Figure 3C). The alteration case numbers, types, and sites of *CALM1* are shown in Figure 3D. We found that the primary genetic alteration of *CALM1* was a missense mutation. R107 was observed in the 3D structure as the most frequent alteration site of *CALM1* (Figure 3E) that could induce translation from R (Arginine) to H (Histidine) or C (Cysteine). However, genetic alterations in *CALM1* barely influenced the OS of cancer patients.

### Immune Infiltration Analysis Data

**FIGURE 4:**
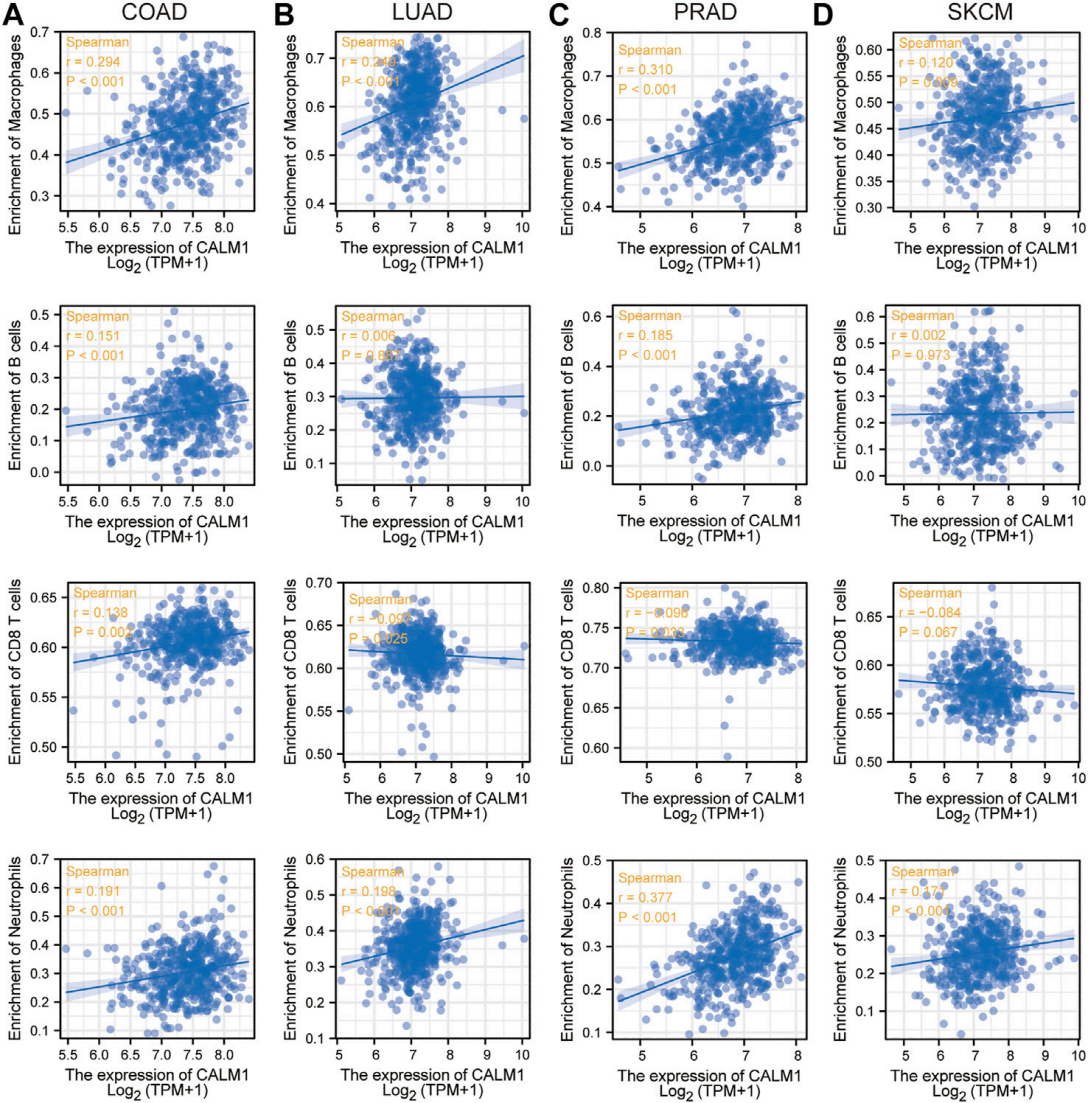
Immune infiltration analysis of *CALM1* expression. Correlation between *CALM1* mRNA levels and immune infiltration in colon adenocarcinoma (COAD) **(A)**, lung adenocarcinoma (LUAD) **(B)**, prostate adenocarcinoma (PRAD) **(C)**, and skin cutaneous melanoma (SKCM) **(D)**.

Previous studies have shown that immune cells in the tumor microenvironment can affect patient prognosis. Therefore, it is meaningful to explore the association between *CALM1* expression and immune infiltration. The results from TCGA indicated a positive relationship between *CALM1* expression and macrophages, B-cells, and neutrophils in COAD, LUAD, PRAD, and SKCM. The number of CD8+T-cells was negatively correlated with *CALM1* expression in LUAD and PRAD, but positively correlated with that in COAD and SKCM (Figure 4). The results from TIMER2 verified *CALM1* expression to be positively correlated with levels of neutrophils and macrophages in four selected cancers (Supplementary Figure S1A). Additionally, we found that different mutant forms of *CALM1* (e.g., arm-level gain, arm-level deletion, and deep deletion) to hamper the infiltration of immune cells (Supplementary Figure S1B).

### Prognostic and Diagnostic Potential Data

**FIGURE 5:**
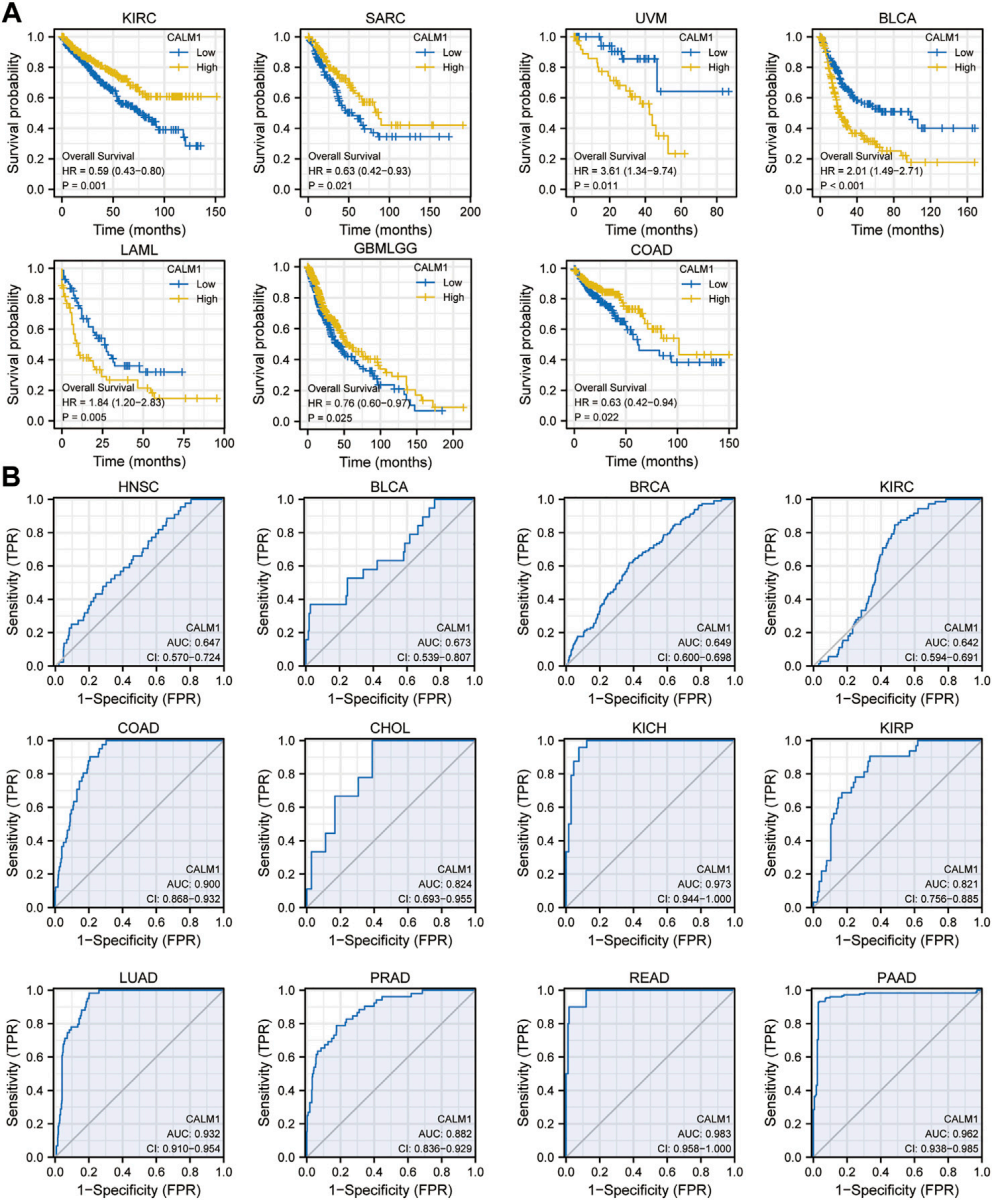
Analysis of prognostic and diagnostic potentials of *CALM1* expression in human cancers. **(A)** Kaplan–Meier survival analysis was conducted to investigate the prognostic value of *CALM1* expression. **(B)** ROC analysis was performed for investigating the diagnostic value of *CALM1* expression. HR, hazard ratio; AUC, area under curve; CI, confidence interval; ROC, receiver operating characteristic.

To evaluate the prognostic and diagnostic potentials of the CALM family genes, we performed KM and diagnostic ROC curve analyses using the data from TCGA. KM survival analysis showed that patients with elevated *CALM1* expression had a favorable OS in KIRC, SARC, glioblastoma multiforme and brain lower-grade glioma (GBMLGG), and COAD, but poorer OS in uveal melanoma (UVM), acute myeloid leukemia (LAML) and BLCA (Figure 5A). The ROC curve demonstrated that *CALM1* expression had a high diagnostic value in COAD, KICH, LUAD, READ, and PAAD (AUC >0.9; Figure 5B). Similarly, *CALM2* and *CALM3* also had high prognostic and diagnostic potentials in human cancers (Supplementary Figure S2, S3). Taken together, the results showed that the CALM family genes could be a promising prognostic and diagnostic biomarker in human cancers.

**FIGURE 6:**
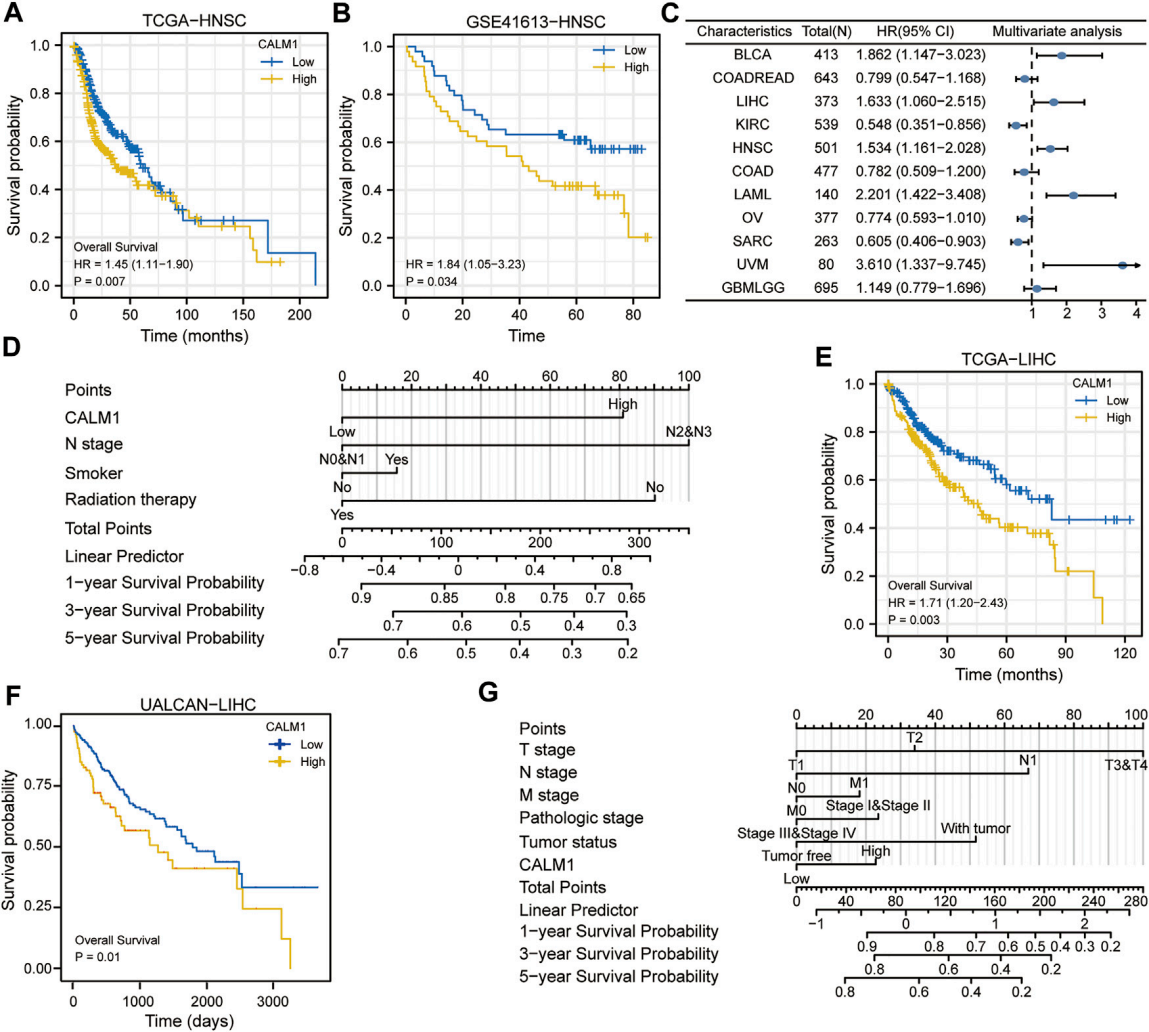
Evaluation prognostic potential of *CALM1* expression and establishment of nomograms in HNSC and LIHC. **(A)** Survival curves show that overall survival was different for patients with low or high expression of *CALM1* in HNSC. **(B)** The GSE41613 dataset was used to verify the prognostic potential of *CALM1* expression in HNSC. **(C)** Forest plot for the prognostic analysis of *CALM1* expression in human cancers. **(D)** Construction of the nomogram model integrating *CALM1* expression, N stage, smoker, and radiation therapy in HNSC. **(E)** Survival analysis show patients with decreased *CALM1* expression had a favorable overall survival in LIHC. **(F)** The UALCAN database was used to verify the prognostic potential of *CALM1* expression in LIHC. **(G)** Construction of a nomogram model integrating *CALM1* expression, TNM stages, pathologic stages, and tumor status in LIHC. HNSC, head and neck squamous cell carcinoma; LIHC, liver hepatocellular carcinoma.

Specifically, in HNSC, patients with decreased *CALM1* expression showed a favorable OS (*P* = 0.007; Figure 6A). KM analysis using data from the GSE41613 dataset confirmed that the low mRNA level of *CALM1* was associated with a favorable OS (*P* = 0.034; Figure 6B). The Cox regression models demonstrated *CALM1* to be an independent risk factors for OS (Figure 6C). Furthermore, we developed a nomogram integrating *CALM1* expression, N stage, smoker, and radiation therapy for predicting the survival probability of HNSC patients (Figure 6D). The calibration curve showed excellent agreement between predicted and actual probabilities at 1, 3, and 5 years with a C-index of 0.625 (range, 0.602–0.649) (Supplementary Figure S4A). The accuracies calculated by AUC in predicting 1, 3 and 5 years OS were 0.662, 0.649, and 0.658, respectively (Supplementary Figure S4B). DCA revealed our nomogram to have a higher net benefit in predicting OS, compared with the traditional TNM model (Supplementary Figure S4C).

To further assess the prognostic predictive value of *CALM1* expression, we selected TCGA-LIHC as the validation set. KM survival analysis confirmed patients with decreased *CALM1* expression had a favorable OS (*P* = 0.003; Figure 6E), which is consistent with the results of UALCAN (*P* = 0.01; Figure 6F). Subsequently, we developed a new nomogram integrating *CALM1* expression, TNM stage, pathologic stage, and tumor status (Figure 6G). The calibration curve showed excellent agreement between predicted and actual probabilities at 1, 3, and 5 years with a C-index of 0.665 (range, 0.630–0.700; Supplementary Figure S4D). The accuracies calculated by AUC in predicting 1, 3 and 5 years OS were 0.688, 0.760, and 0.761, respectively (Supplementary Figure S4E). However, DCA revealed that the traditional TNM model has a higher net benefit in predicting OS, compared with our nomogram (Supplementary Figure S4F). Taken together, *CALM1* mRNA levels had good applicability and can not only predict the prognosis of HNSC but also serve as a prognostic predictive biomarker for LIHC.

### Insights Into *CALM1* Function

**FIGURE 7:**
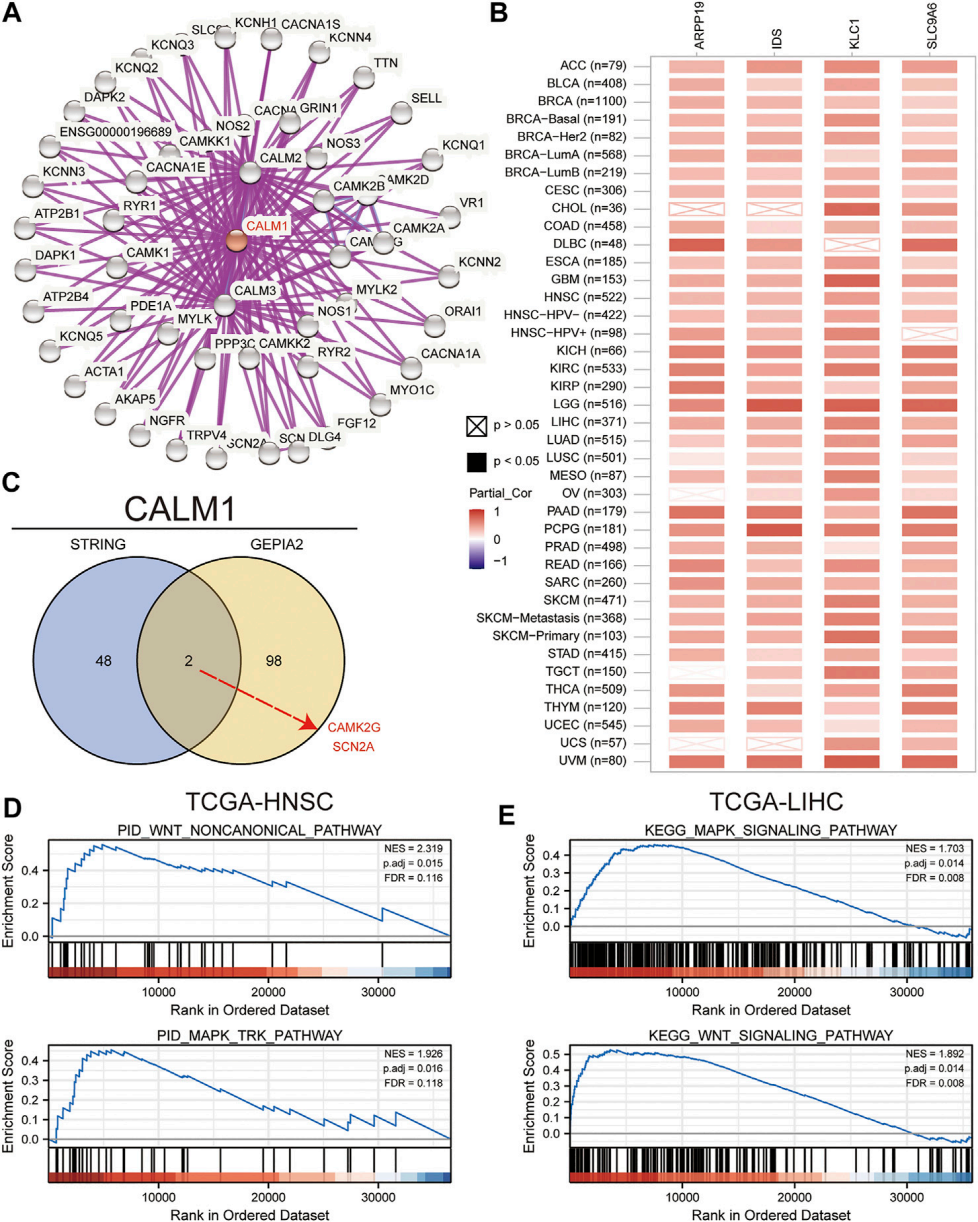
Insights into *CALM1* function. **(A)** We obtained the protein-protein interaction network of *CALM1* using the STRING website. **(B)** We analyzed the pan-cancer correlation between *CALM1* expression and *SLC9A6*, *ARPP19*, *KLC1*, and *IDS* using the TIMER2 website. **(C)** We conducted an intersection analysis for the *CALM1*-binding and *CALM1*-correlated genes. Finally, we performed GSEA to elucidate functional differences for *CALM1*-high expression group versus *CALM1*-low expression group in HNSC **(D)** and LIHC **(E)**. *SLC9A6*, solute carrier family 9-member a6; *ARPP19*, CAMP regulated phosphoprotein; *KLC1*, kinesin light chain 1; *IDS*, iduronate 2-sulfatase; *CAMK2G*, calcium/calmodulin dependent protein kinase II gamma; *SCN2A*, sodium voltage-gated channel alpha subunit 2; GSEA, Gene Set Enrichment Analysis; TCGA, The Cancer Genome Atlas; HNSC, head and neck squamous cell carcinoma; LIHC, liver hepatocellular carcinoma.

To understand the mechanism underlying the role of *CALM1* in tumorigenesis, we conducted KEGG and GO enrichment analyses using 50 *CALM1*-binding proteins and 100 *CALM1* expression-correlated genes. In total, 50 *CALM1*-binding proteins were identified using the STRING database, which was further verified experimentally. The interaction network of these proteins is presented in Figure 7A. A total of 100 *CALM1* expression-correlated genes were identified using the GEPIA2 database. Among them, *SLC9A6, KLC1, ARPP19,* and *IDS* were significantly related to *CALM1* overexpression, according to the data from the TIMER2 database (Figure 7B). The intersection analysis integrating 50 *CALM1*-binding proteins and 100 *CALM1* expression-correlated genes showed two common members, namely *CAMK2G* and *SCN2A* (Figure 7C). Finally, we conducted GSEA to explore the roles of *CALM1* in cancers. We found that *WNT* and *MAPK* signaling pathways associated gene sets were significantly enriched in the *CALM1* high expression group in both HNSC (Figure 7D) and LIHC (Figure 7E). Other cancer-related gene sets, such as the *PI3K-Akt* and *Hippo* signaling pathways, were found to be affected by different *CALM1* expression levels (Supplementary Figure S5).

## Discussion

As the major Ca^2+^ sensor, CALM, encoded by the *CALM1* gene in mammals, is highly conserved in eukaryotic cells. Previous studies had found *CALM1* to be upregulated in nasopharyngeal carcinoma, prostate cancer, and bladder cancer, and had reported its oncogenic role in esophageal squamous cell carcinoma. However, little is known regarding the global function and expression landscape of *CALM1* in other cancers. In this report, we comprehensively assessed the *CALM1* expression pattern based on TCGA data. The results demonstrated that *CALM1* levels are increased in CESC, DLBC, HNSC, LAML, LIHC, OV, PAAD, PCPG, SKCM, STAD, TGCT, UCEC, and THYM, but decreased in COAD, KICH, LGG, LUAD, PRAD, and READ, compared with that in normal tissues. Further validation using the HPA databases revealed that the protein expression of *CALM1* increases in breast cancer, liver cancer, and pancreatic cancer, but decreases in colorectal cancer, prostate cancer, and lung cancer, compared with that in normal tissues. The differential expression profiles might reflect the distinct molecular functions of *CALM1* in human cancers.

Since cancer progression involves a series of abnormal regulations affected by gene alterations (Liu et al., 2021) or DNA methylation (Liu et al., 2021), we aimed to investigate the potential correlation across gene alterations, DNA methylation, and patient survival. We observed several genetic alterations of *CALM1*, including deep deletion, amplification, and missense mutation; although these alterations might affect cancer progression, according to the data from the cBioPortal database, they did not affect prognostic outcomes. Additionally, we found *CALM1* methylation to be significantly increased in CESC and COAD compared with normal tissues and significantly decreased in LIHC and PCPG, showing an inverse trend compared with that of the expression pattern. This finding implies that *CALM1* expression is regulated by DNA methylation in human cancers. Subsequently, we found a negative association between DNA methylation and *CALM1* expression in CESC, COAD, LIHC, and PCPG, which further demonstrates the regulatory ability of DNA methylation on *CALM1* expression. The results collectively suggested that *CALM1* methylation plays a significant and complicated role in human cancers; however, more in-depth research is needed to verify this conclusion.

Previous studies had found immune cell infiltration to significantly affect cancer progression and prognosis (Liu et al., 2017). M1 macrophages are known to inhibit cancer progression, CD4+T-cells can recognize cancer antigens (Lin et al., 2019), CD8+T-cells can inhibit cancer metastasis (Joseph et al., 2021), and immune infiltration of T-cells can significantly influence the efficacy of immunotherapy (Borst et al., 2018). This study found a statistically significant positive correlation between immune infiltration and *CALM1* expression in human cancers. The different *CALM1* mutational forms hampered immune infiltration. The results collectively imply that *CALM1* plays an essential role in the regulation and recruitment of immune cell infiltration, which might eventually affect patient prognostic outcomes.

Another key finding of this study is that *CALM1* expression can indicate different prognostic outcomes in human cancers. Kaplan–Meier survival analysis showed that patients with elevated *CALM1* expression had a favorable OS in KIRC, SARC, GBMLGG, and COAD, but poorer OS in UVM, LAML, LIHC, and BLCA. The multivariate Cox model further confirmed *CALM1* expression as an independent risk factor for OS in human cancers. Additionally, we constructed a nomogram integrating clinical variables and *CALM1* expression for predicting survival rate in HNSC patients, and it performed better than the traditional TNM model. The ROC curve demonstrated that *CALM1* expression had a high diagnostic value in COAD, KICH, LUAD, READ, and PAAD (AUC >0.9). Overall, the results suggest that *CALM1* could be a valuable prognostic and diagnostic biomarker in human cancers.

We performed GSEA to predict the potential molecular function of *CALM1* in cancers. A previous study analyzing the hyperglycemia of obese diabetic mice had revealed that elevated *CALM1* expression can directly activate the *PI3K-Akt* pathway to repress gluconeogenic gene expression in hepatocytes (Chen et al., 2017). Consistent with this report, our functional enrichment analysis showed that increased *CALM1* expression could activate the *PI3K-Akt* signaling pathway. This pathway involves cell apoptosis, oxidative stress, and inflammation, and plays a vital regulatory role in various malignant tumors. Our results show that increased *CALM1* expression could also activate other cancer-related pathways, such as the *Hippo, Wnt*, and *MAPK* signaling pathways. Previous studies had revealed that the activated *Hippo* pathway increases ovarian cancer stemness and tumor resistance (Muñoz-Galván et al., 2020). The activated *Wnt* pathway promotes cell growth and migration in squamous cell lung carcinoma (Wu et al., 2021). Similarly, the activated *MAPK* pathway regulates various cellular functions, such as apoptosis, survival, differentiation, and proliferation (Liu et al., 2018). The results overall suggest that *CALM1* is closely associated with cancer progression.

This study has some limitations. First, no experimental validation was performed. Second, the correlation between mRNA levels and protein expression of *CALM1* would need further verification. Third, since *CALM1* plays a very complex role in cancer prognosis, we could not define the exact role of *CALM1* as either oncogenic or protective.

## Conclusion

Our study was the first to elucidate the expression landscape, DNA methylation, gene alteration, immune infiltration, clinical relevance, and molecular pathways of *CALM1* in multiple cancers using in-silico multi-omics analysis. *CALM1* was found to be differentially expressed in human cancers and adjacent normal tissues, with a low mutation frequency. DNA methylation of *CALM1* regulated its expression in multiple cancers. *CALM1* expression was closely related to immune infiltration and cancer-related cellular pathways. Additionally, *CALM1* expression had a high diagnostic and prognostic potential in human cancers. These findings collectively offer a relatively comprehensive understanding of the functional significance of *CALM1* in human cancers.

## Data Availability

The original contributions presented in the study are included in the article/Supplementary Material, further inquiries can be directed to the corresponding authors.
