# Supplementary material for: In-Silico Multi-Omics Analysis of the Functional Significance of Calmodulin 1 in Multiple Cancers
Source: Front Genet. 2022 Jan 12;12:793508. doi: 10.3389/fgene.2021.793508 (PMC8790318; doi:10.3389/fgene.2021.793508)
Supplement: Supplementary file 1 [file DataSheet1.docx]

Supplementary Material


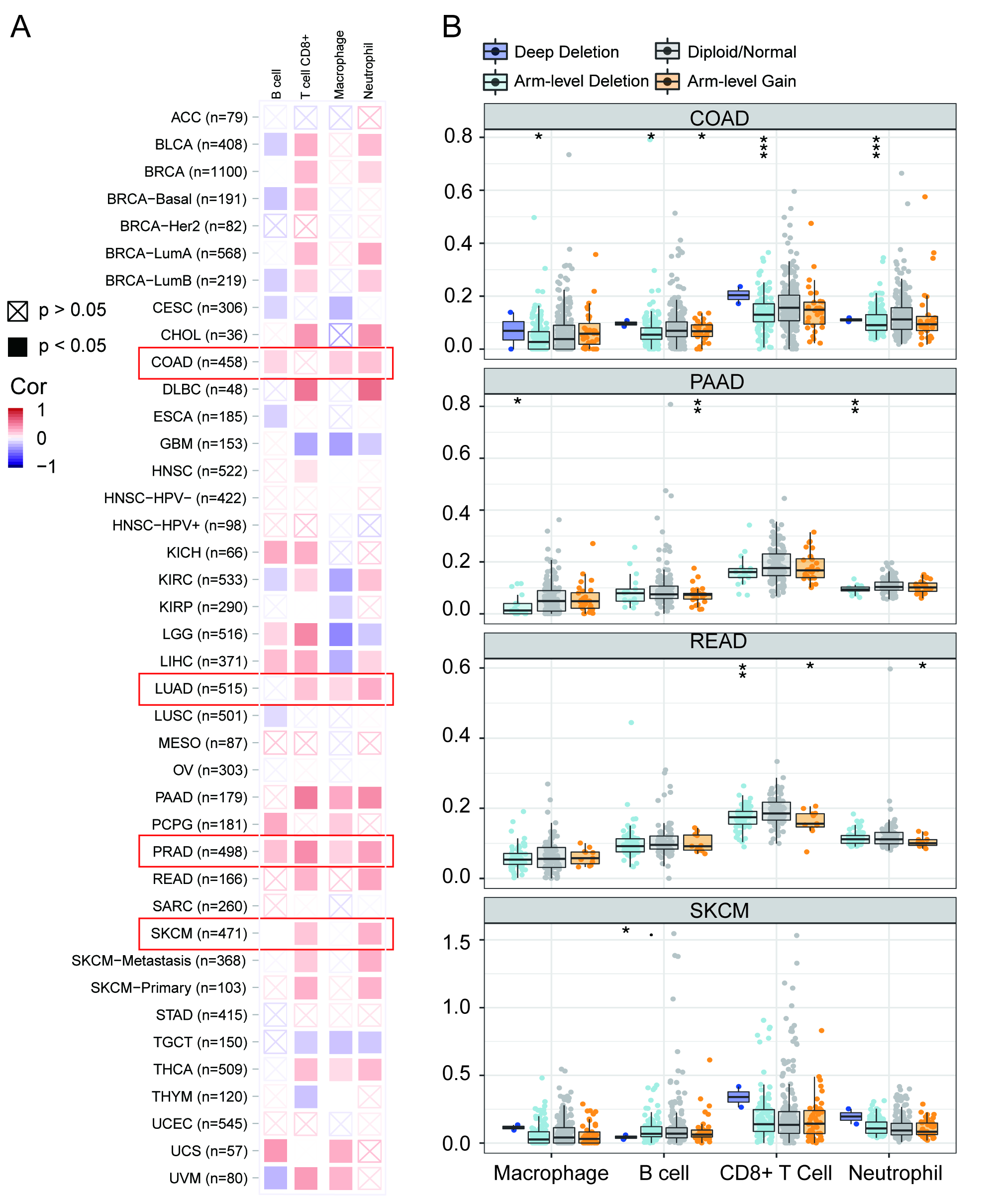


Fig. S1. Immune infiltration analysis of *CALM1* expression. (A) The correlation between *CALM1* mRNA expression and immune infiltration. The four cancers in the red box showed relatively consistent positive correlations compared with other cancer types. B. The comparison of cancer infiltrating levels in four cancers with different alterations for *CALM1* from the TIMER database. These alterations included deep deletion, Arm-level deletion, Diploid, and Arm-level gain. The immune cells included B cell, CD8+T cell, Macrophages, and Neutrophil. **P*<0.05, ***P*<0.01, ****P*<0.001.


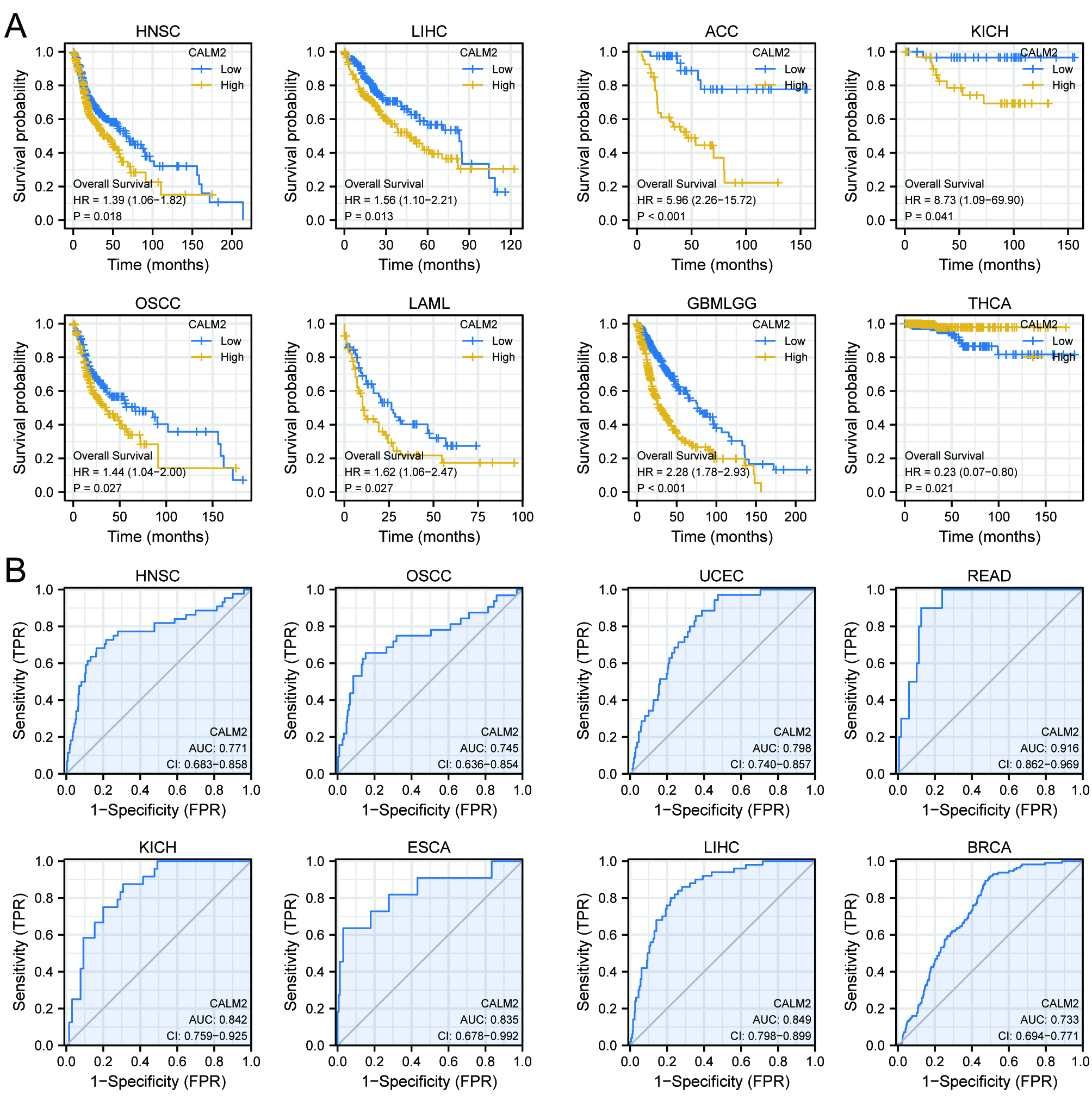


Fig. S2. Prognosis and diagnosis analysis of *CALM2* expression in human cancers. (A) Kaplan-Meier survival analysis was conducted to investigate the prognostic value of *CALM2* expression. B. ROC analysis was performed for investigating the diagnostic value of *CALM2* expression. HR, hazard ratio; AUC, area under curve; CI, confidence interval; ROC, receiver operating characteristic.


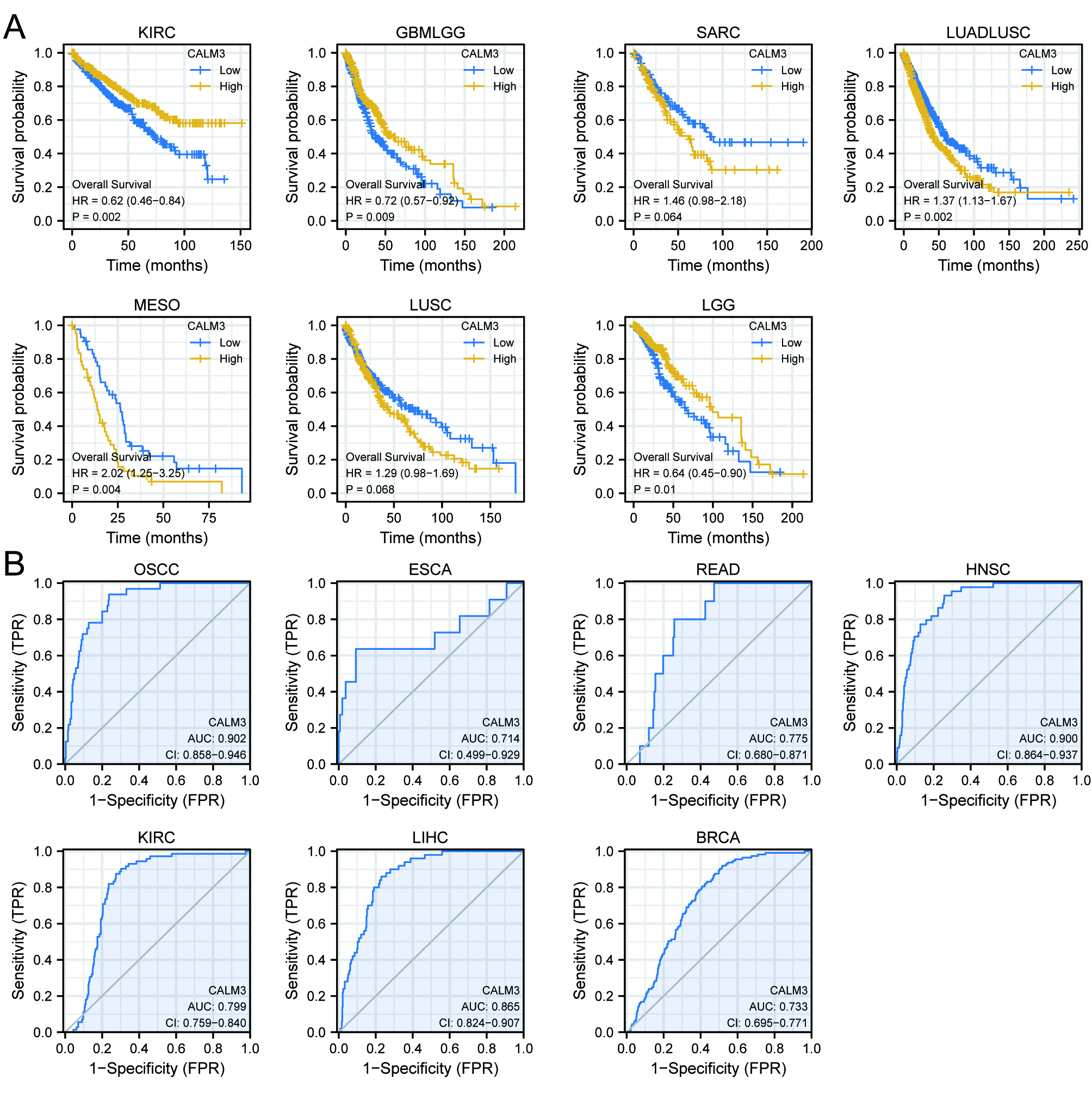


Fig. S3. Prognosis and diagnosis analysis of *CALM3* expression in human cancers. (A) Kaplan-Meier survival analysis was conducted to investigate the prognostic value of *CALM3* expression. B. ROC analysis was performed for investigating the diagnostic value of *CALM3* expression. HR, hazard ratio; AUC, area under curve; CI, confidence interval; ROC, receiver operating characteristic.


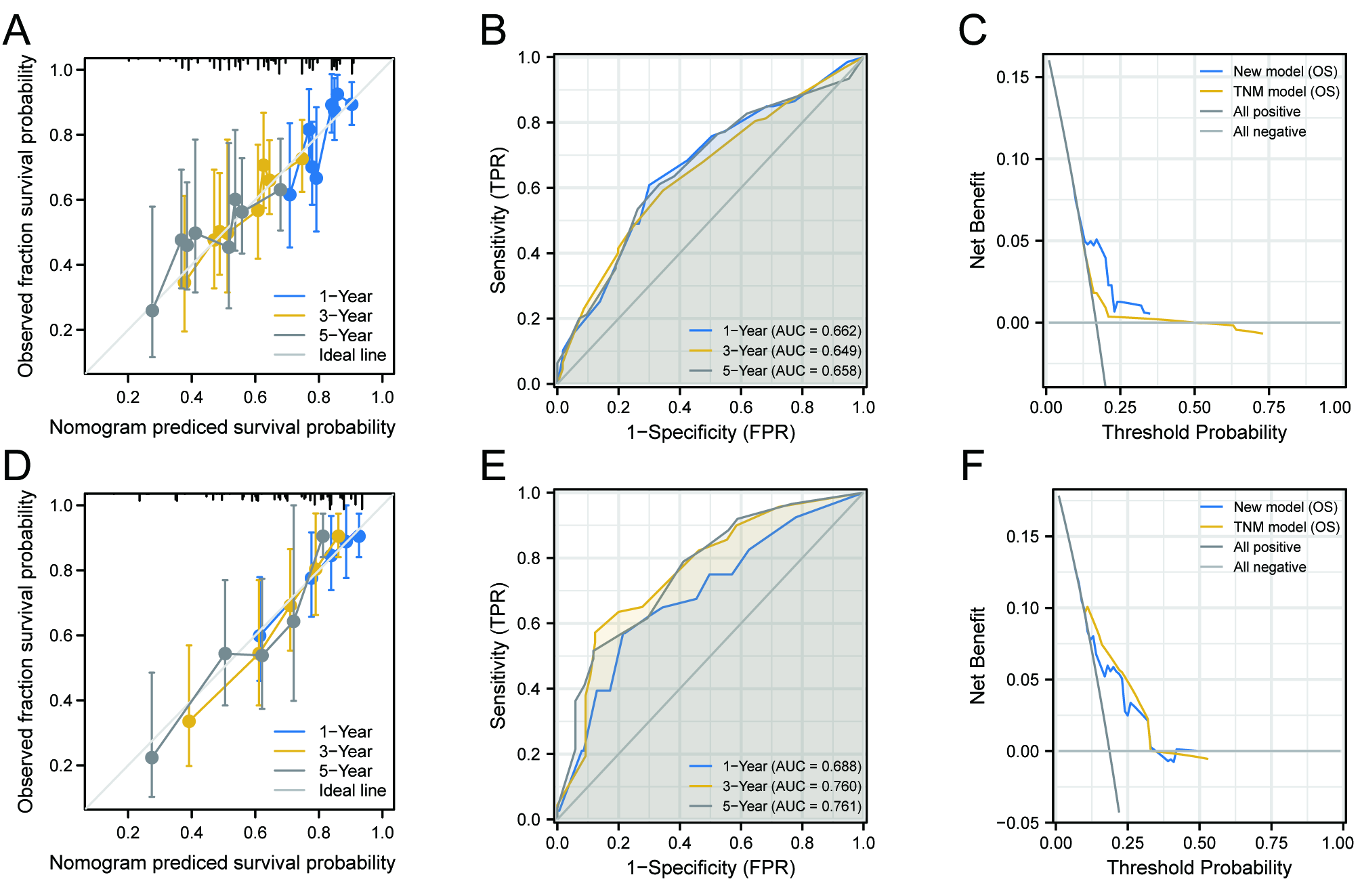


Fig. S4. Evaluation of nomograms in HNSC and LIHC. Calibration curve (A), ROC curve (B), and DCA (C) were used to evaluate the predictive accuracy, discrimination ability, and clinical utility of the nomogram model in TCGA-HNSC, respectively. Similarly, Calibration curve (A), ROC curve (B), and DCA (C) were used to evaluate the predictive accuracy, discrimination ability, and clinical utility of the nomogram model in TCGA-LIHC, respectively. ROC, receiver operating characteristic; DCA, decision curve analysis. HNSC, head and neck squamous cell carcinoma. LIHC, liver hepatocellular carcinoma.


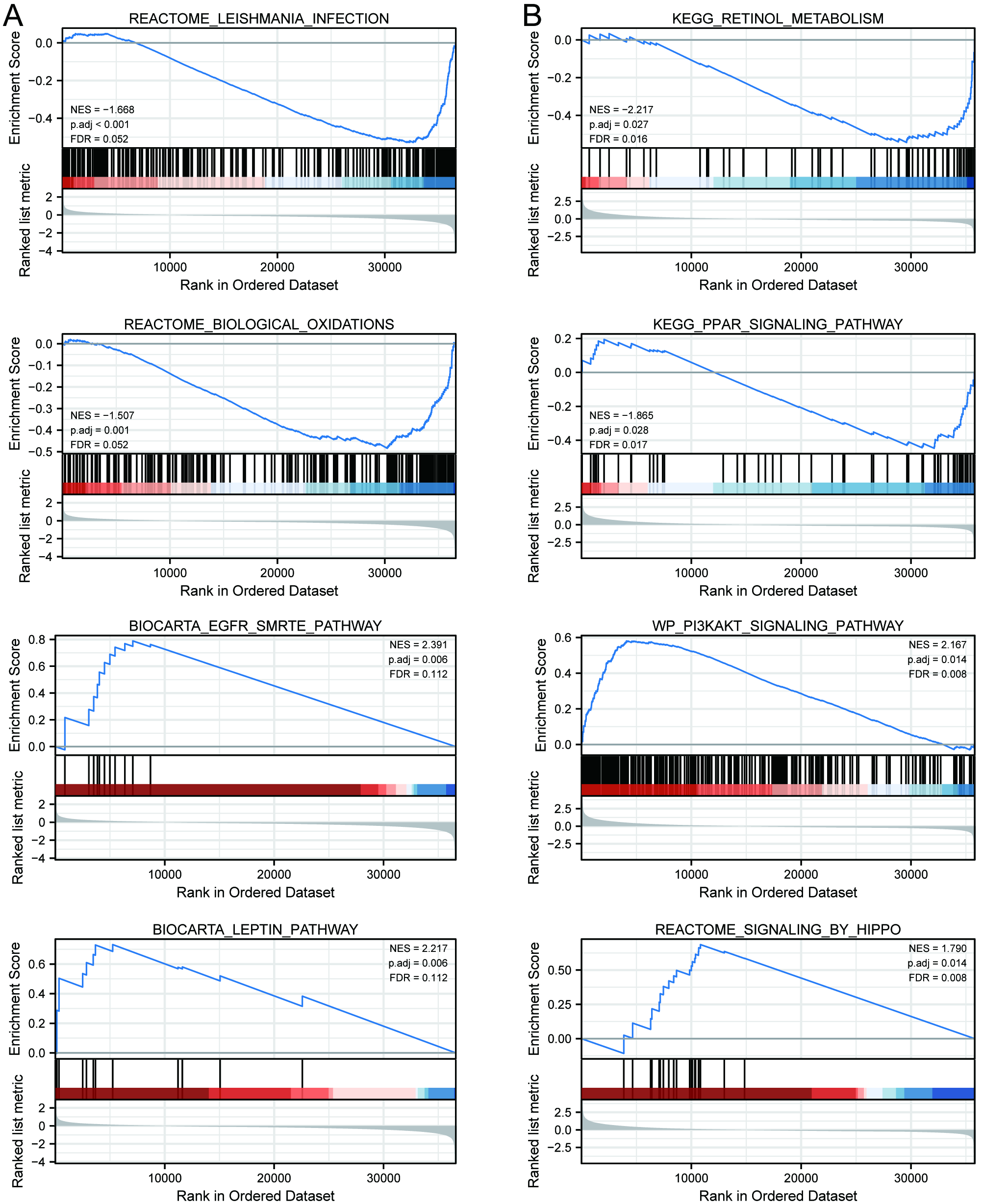


Fig. S5. *CALM1*-related signaling pathways based on GSEA. Typical results of the GSEA for a single gene set in HNSC (A) and LIHC (B). NES, normalized ES; p.adj, adjust *p* value; FDR, false discovery rate. HNSC, head and neck squamous cell carcinoma; LIHC, liver hepatocellular carcinoma.

Table S1. Detailed information of TCGA cancer types

| Cancer type | Full name |
| --- | --- |
| ACC | adrenocortical cancer |
| BLCA | bladder urothelial carcinoma |
| BRCA | breast invasive carcinoma |
| CESC | cervical and endocervical cancer |
| CHOL | cholangiocarcinoma |
| COAD | colon adenocarcinoma |
| DLBC | diffuse large B-cell lymphoma |
| ESCA | esophageal carcinoma |
| GBM | glioblastoma multiforme |
| HNSC | head and neck squamous cell carcinoma |
| KICH | kidney chromophobe |
| KIRC | kidney clear cell carcinoma |
| KIRP | kidney papillary cell carcinoma |
| LGG | brain lower grade glioma |
| LIHC | liver hepatocellular carcinoma |
| LUAD | lung adenocarcinoma |
| LUSC | lung squamous cell carcinoma |
| MESO | mesothelioma |
| OV | ovarian serous cystadenocarcinoma |
| PAAD | pancreatic adenocarcinoma |
| PCPG | pheochromocytoma and paraganglioma |
| PRAD | prostate adenocarcinoma |
| READ | rectum adenocarcinoma |
| SARC | sarcoma |
| SKCM | skin cutaneous melanoma |
| STAD | stomach adenocarcinoma |
| TGCT | testicular germ cell tumor |
| THCA | thyroid carcinoma |
| THYM | thymoma |
| UCEC | uterine corpus endometrioid carcinoma |
| UCS | uterine carcinosarcoma |
| UVM | uveal melanoma |
| LAML | acute myeloid leukemia |
